# Risk of complications in patients with one eye during and after ocular surgery

**DOI:** 10.22336/rjo.2024.74

**Published:** 2024

**Authors:** Tugce Horozoglu Ceran, Mehmet Citirik, Mehmet Yasin Teke

**Affiliations:** 1 Ministry of Health, Osmaniye State Hospital, Osmaniye, Turkey; 2 University of Health Sciences, Ankara Etlik City Hospital, Ankara, Turkey

**Keywords:** surgical complication, phacoemulsification, cataract, vitreoretinal surgery, legal blindness, one-eyed, AMD = Age-related macular degeneration

## Abstract

**Purpose:**

This study examined patients with permanent legal blindness in one eye after a previous eye surgery and those with permanent legal blindness in one eye for non-surgical reasons. The objective of this study was to assess the occurrence of complications both during and after surgery in patients undergoing phacoemulsification for cataracts or vitreoretinal surgery for retinal detachment in the fellow eye.

**Methods:**

A retrospective study included 62 patients (group 1) with legal blindness in one eye, compared to 62 control patients (group 2) undergoing similar surgeries. Complications, history of complex surgery leading to legal blindness, and demographic characteristics were analyzed.

**Results:**

In group 1, the complication rate was significantly higher in patients who underwent both phacoemulsification and vitreoretinal surgeries than in group 2 (p < 0.05). In group 1, complications developed during surgery in the other eye in 28.1% of the patients whose permanent legal blindness stemmed from a complication in the previous surgery. In contrast, complications arose in the surgery of the other eye in 10% of patients whose permanent legal blindness did not result from any complications in the previous surgery. A statistically significant difference was observed between the two groups (P < 0.05).

**Discussion:**

In our study, if the cause of eye loss in patients with permanent legal blindness in one eye was a surgical complication, the possibility of complications during surgery in the other eye was high. Surgery can be planned at higher visual acuity levels in a patient who has lost one eye for reasons other than surgery. In patients who have lost one eye due to previous surgery, surgery for the other can be planned at lower visual acuity levels.

**Conclusion:**

This is the first report to compare the rate of complications during and after surgery in patients with pre-existing permanent legal blindness in one eye who underwent cataract surgery and vitreoretinal surgery in the other.

## Introduction

Blindness and poor vision are major health problems that cause social and economic issues worldwide. Blindness is defined as less than 10 percent vision or a field of vision narrower than 20° in a person’s eye despite correction with glasses or contact lenses. World Health Organization data shows 285 million people have vision problems [1]. Approximately 45 million people live legally blind [2]. Owing to population aging and growth, this number is increasing by one million almost every year[3,4]. The most common causes of blindness are cataracts, uncorrected refractive disorders, and age-related macular degeneration [5]. According to data from European countries, age-related macular degeneration (AMD), glaucoma, and diabetic retinopathy are the most common causes of blindness [6]. In Europe, AMD has been reported as the most common cause of blindness in older age groups and diabetic retinopathy in working-age groups [7]. Very few studies have investigated the causes and prevalence of blindness in Turkey [8].

When phacoemulsification or vitreoretinal surgery is indicated for cataracts or vitreoretinal diseases in the other eye of patients with permanent blindness in one eye without curable causes, patients/physicians have hesitations and fears regarding the surgical approach to their eyes with good vision due to blindness in one eye. Physicians face a dilemma while operating on patients’ eyes for treatable reasons. On the one hand, there is uncertainty about whether to proceed with surgery when the disease has not progressed significantly and visual acuity has not decreased substantially, aiming to minimize the risk of complications. However, the question arises about whether to operate when visual acuity affects the patient’s quality of life. While some studies have reported that early surgery is beneficial, others have not supported this information [9-11].

In the case of a pathology requiring a surgical approach to the other eye of patients with blindness in one eye, if complications develop during surgery, there is a fear of decreased vision in the well-sighted eye. These patients can be referred to more advanced centers regardless of the surgical need for their well-sighted eyes and the reason for the loss of the blind eye. The main reason for this situation is the belief that in surgical complications, these patients should be operated on by physicians and hospitals competent to intervene during surgery. Another reason is the protective approach of surgeons to avoid medicolegal problems if a loss develops in the patient’s other eye. Intervention in the other eye of a patient with blindness in one eye creates psychological stress and performance anxiety among ophthalmologists [1-8]. It is unclear whether the primary pathology of the eyes of patients with legal blindness in one eye increases the likelihood of complications during surgery if the other eye requires surgery [8-11].

This study investigated individuals with permanent legal blindness following eye surgery and those with permanent legal blindness in one eye due to non-operative causes. The assessment included examining complications in the other eye during and after surgery in patients undergoing phacoemulsification surgery for cataracts or vitreoretinal surgery for retinal detachment.

## Methods

This retrospective observational study was conducted according to the Declaration of Helsinki. The Health Sciences University Ankara Health Practice and Research Center provided Ethics Committee approval.

The study included patients with permanent blindness in one eye who were treated at the Health Sciences University Ankara Ulucanlar Eye Health Application and Research Center between January 2020 and December 2021 and those with blindness in one eye who underwent phacoemulsification or vitreoretinal surgery. Patients who did not have permanent blindness in either eye but underwent vitreoretinal surgery due to phacoemulsification or retinal detachment were included in the control group.

The patients’ files were examined, and age, sex, diagnosis, cause of blindness in one eye, the procedure of the surgery in the seeing eye, and whether complications developed during the seeing eye surgery were noted. Patients in the working age group (18-65 years) were included in this study.

Patients with an indication for cataract surgery, whose history of visual acuity limited their daily life, and whose visual acuity was 20/200-20/50 were operated on. The Centurion® (Alcon, Texas, USA) ultrasonic phacoemulsification device was used by the same surgeon with 20 years of experience in cataract surgery. Topical anesthesia was administered to all patients who underwent cataract surgery. All patients undergoing cataract surgery have had the same type of hydrophobic intraocular lens implanted. In the surgical notes recorded in the patient’s files, posterior capsule opening during phacoemulsification surgery, the transition of the nucleus into the vitreous, zonular dialysis, vitreous transition to the anterior chamber, need for anterior vitrectomy, iris prolapse, need for lens implantation into the ciliary sulcus/anterior chamber/sclera, pseudophakic bullous keratitis, intraocular lens dislocation, pseudophakic macular edema, posterior capsule opacification, visual acuity that did not increase after the postoperative 6th month, increased intraocular pressure at the postoperative 6th month, vitreous hemorrhage, suprachoroidal hemorrhage, retinal detachment, and the presence of endophthalmitis were evaluated as complications, and their presence was recorded.

Patients with vitreoretinal surgery indications were diagnosed with rhegmatogenous retinal detachment involving the macula, and patients with a visual acuity of 20/200 to 20/1250 underwent surgery. Among the cases, those with grade C1 and C2 proliferative vitreoretinopathy were included in the study, while those who were more advanced were not. Vitreoretinal surgery was performed by the same surgeon, with 20 years of experience, using the Constellation Vitrectomy® (Alcon, Fort Worth, Texas, USA) 25+ system. A retrobulbar anesthetic injection was administered to patients who underwent vitreoretinal surgery. An iatrogenic tear during vitreoretinal surgery, need for retinotomy, subretinal hemorrhage, visual acuity that did not increase after 6 months postoperatively, increased intraocular pressure at 6 months postoperatively, hypotonia, cystoid macular edema, epiretinal membrane development, recurrent retinal detachment failure), proliferative vitreoretinopathy, the conditions mentioned above due to phacoemulsification, the presence of endophthalmitis, and the development of phthisis bulbi were evaluated as complications and their presence was recorded.

Statistical analysis was performed using SPSS 23.0 (SPSS Inc., Chicago, IL, USA). Minimum and maximum values, mean, standard deviation, number, and percentage (%) were analyzed for descriptive statistics. The Shapiro-Wilk test was used to evaluate the normality of the distribution. Pearson Chi-Square and Fisher precision tests were used to evaluate the categorical variables between and within the groups. Statistical significance was set at P < 0.05.

## Results

Sixty-two patients with permanent legal blindness in one eye (group 1) and 62 patients who underwent cataract surgery or vitreoretinal surgery without legal blindness in any eye (group 2) were included in the study.

In group 1, 27 patients (43.5%) underwent phacoemulsification surgery for cataracts, and vitreoretinal surgery was performed in 35 patients (53.5%) for retinal detachment. In group 2, 25 patients (40.3%) underwent phacoemulsification surgery for cataracts, and vitreoretinal surgery was performed in 37 patients (59.7%) for retinal detachment.

Table 1 shows the causes of vision loss in patients in group 1 who had and did not have complications related to previous surgery due to preoperative permanent legal blindness in the other eye.

**Table 1:** Causes of visual loss in patients with and without complications from previous surgery and the cause of permanent legal blindness before surgery in the other eye

	Diagnosis	Number	Percent (%)
**Permanent legal blindness before surgery without complications from previous surgery**	Diabetic retinopathy	10	33.3
	Age-related macular degeneration	7	23.3
	Corneal leukoma	6	20
	Amblyopia	5	16.7
	Glaucoma	2	6.7
	Total	30	100
**Permanent legal blindness before surgery due to complications from previous surgery**	Past retinal detachment surgery	13	40.6
	Past cataract surgery	8	25
	Prior penetrating injury surgery	7	21.9
	Prior glaucoma surgery	3	9.4
	Previous penetrating keratoplasty surgery	1	3.1
	Total	32	100

In group 1, 51.6 % (32/62) had permanent legal blindness due to surgical complications in the other eye, while 48.4% (30/62) had permanent legal blindness without surgical complications. In group 1, phacoemulsification was performed in 14 patients (43.8%), and vitreoretinal surgery was performed in 18 (56.2%) of 32 patients with permanent legal blindness due to surgical complications in the other eye.

Table 2 presents the demographic data of the groups. The mean age was 53.2 ± 1.29 (20-65) years in group 1 and 53.1 ± 2.52 (18-65) years in the control group. 66.1% (41/62) of group 1 patients were male, and 72.6% (45/62) of group 2 patients were male. There were no significant differences between the two groups regarding age or sex (P > 0.05).

**Table 2:** Demographic data of the groups

		Group 1	Group 2
Number of eyes		62	62
Mean age ± standard deviation (years)/range		53,2 ± 1,29 (20-65)	53,1 ± 2,52 (18-65)
Gender	Male	41	45
	Female	21	17

In group 1, the complication rate of patients who underwent phacoemulsification surgery was 14.8% (4/27). The most common complications were posterior capsule opacification, rupture, and vitreous loss. In group 2, the surgical complication rate of patients who underwent phacoemulsification surgery for cataracts was 4% (1/25), and posterior capsule opacification was the most common finding. A statistically significant difference was identified between the two groups regarding complications during the surgical procedures (p=0.015).

In group 1, the complication rate of patients who were permanently legally blind due to a surgical complication in the other eye and underwent phacoemulsification surgery was 21.4% (3/14); the most common complications were posterior capsule rupture and vitreous loss. The complication rate in patients who were permanently legally blind without any surgical complications in the other eye was 7.7% (1/13), with the most common complication being posterior capsule opacification. A statistically significant difference was observed between the two groups (P < 0.001).

Among the patients in group 1, 15 (42.9%, 15/35) had grade C1 proliferative vitreoretinopathy, while 20 patients (57.1%, 20/35) had grade C2 proliferative vitreoretinopathy. Among the patients in group 2, 16 (43.2%, 16/37) had grade C1 proliferative vitreoretinopathy, while 21 patients (56.8%, 21/37) had grade C2 proliferative vitreoretinopathy. There was no significant difference between the two groups regarding proliferative vitreoretinopathy (p > 0.05).

In group 1, the complication rate of patients who underwent vitreoretinal surgery for retinal detachment was 22.8% (8/35). The most common complications were increased intraocular pressure, cystoid macular edema, and recurrent retinal detachment. In group 2, 10.8% (4/37) of the patients who underwent vitreoretinal surgery for retinal detachment had complications following surgery of their eyes, and increased intraocular pressure and epiretinal membrane development were the leading causes. There was a statistically significant difference between the two groups in the development of complications during surgery (p=0.017).

In group 1, the complication rate of patients who were permanently legally blind due to a surgical complication in the other eye and who underwent vitreoretinal surgery was 33.3% (6/18), and the most common complications were increased intraocular pressure and recurrent retinal detachment. The complication rate of patients who were permanently legally blind without any surgical complications in the other eye was 11.7% (1/17), and the most common complication was increased intraocular pressure. A statistically significant difference was observed between the two groups (P < 0.001).

In group 1, 28.1% (9/32) of the patients whose cause of permanent legal blindness was a complication of a previous surgery also developed complications during surgery of the other eye. In group 1, 10% (3/30) of the patients whose cause of permanent legal blindness was not the result of a previous surgery developed complications during surgery of the other eye (Table 3). A statistically significant difference was observed between the two groups (P < 0.001).

**Table 3:** The surgeries applied to the cases and the incidence of complications

			Number of Cases		Complication Rate		Total Complication Rate		P value 1	P value 2
			n	%	n	%	n	%		
**Group 1**	**Group 1a**	Cataract Surgery	14	43.8	3	21.4	9	28.1	0.974	0.716
		Vitreoretinal Surgery	18	56.2	6	33.3				
	**Group 1b**	Cataract Surgery	13	43.3	1	7.7	3	10		
		Vitreoretinal Surgery	17	56.7	2	11.7				
**Group 2**		Cataract Surgery	25	40.3	1	4	5	8	n.a	
		Vitreoretinal Surgery	37	59.7	4	10.8				

**Group 1**: Cases with permanent legal blindness in one eye **Group 1a**: Permanent legal blindness due to complications from previous surgery **Group 1b**: Permanent legal blindness without complications from previous surgery **Group 2**: Cases without legal blindness in any eye **P value 1**: P value between Group 1a and 1b **P value 2:** P value between Group 1 and 2

## Discussion

While age-related macular degeneration is the most common cause of blindness in adults, diabetic retinopathy is considered to occur in the working age group [5]. In our study, diabetic retinopathy was the most common cause of nonsurgical blindness in one eye, and age-related macular degeneration was the second most common cause (**Table 1**).

In the case of a cataract diagnosis in the other eye of patients with permanent legal blindness in one eye, phacoemulsification surgery was performed. Cataract surgery is generally indicated when a decrease in visual acuity is detected, which affects patients’ daily lives. There is no consensus on the ideality of this limit in patients with permanent legal blindness in one eye. For this reason, studies have been conducted to determine the ideal operation time for cataract patients and the benefit-risk ratio. Patients who have lost one eye due to a surgical complication may be tempted to postpone surgery to their sighted eye because of their fear of losing the other eye [9-13].

In their study, Zhu et al. showed that surgery is more beneficial in patients with permanent legal blindness in one eye with a visual acuity of 20/40 and above since it increases the patient’s quality of life [9]. Similarly, Amesbury et al. presented data supporting this opinion in their study [10]. A previous study showed that good preoperative visual acuity does not affect postoperative visual acuity in cataract surgery patients [11]. In contrast, another study showed that patients with good preoperative visual acuity had better outcomes [12].

In a study from Switzerland, the rate of development of capsular complications and the risk factors for capsular complications were evaluated over eight years. The intraoperative posterior capsule opening risk was found to be higher in patients with preoperative low corrected visual acuity (≤ 0.1), glaucoma, and diabetic retinopathy [13]. After eight years, the rate of development of capsule complications and the number of patients with risk factors have decreased [13]. The decrease in the rate of development of capsule complications may be the decrease in the number of patients with risk factors or an increase in surgical experience [13]. In our study, posterior capsule opacification was most common in both groups who underwent phacoemulsification surgery, and posterior capsule rupture and vitreous loss were high in group 1.

In one study, the complication rate was 5.05% during phacoemulsification surgery in one-eye patients [12]. This rate was lower than the rate of 14.8% in our study, which may be because 32% of the patients in the study by Charles et al. had glaucoma, 22% had AMD, and 24% had amblyopia [12]. In our study, 51.6% (32/62) of the patients in group 1 had permanent legal blindness in the other eye due to surgical complications, while 48.4% (30/62) had permanent legal blindness without surgical complications. When cases of permanent legal blindness without surgical complications were evaluated alone, the rate was 7.7%.

The rate of primary retinal reattachment in cases with primary rhegmatogenous retinal detachment (retinal tear and retinal detachment due to posterior vitreous detachment) is 85-90% [14,15]; it has been reported as 60-70% in high-risk eyes (retinal detachment other than primary rhegmatogenous retinal detachment) [16]. Functional or anatomical success cannot be achieved in approximately 5% of eyes [17]. In a regional study conducted in Ireland, the primary success rate after vitreoretinal surgery was 86 % [18]. In the literature, when evaluating the success and postoperative complication rates of vitreoretinal surgeries, cases of primary rhegmatogenous retinal detachment and cases of proliferative vitreoretinopathy were assessed separately. In our study, we selected patients with stage C1 and C2 proliferative vitreoretinopathy in the control and study groups, and we observed similar results to those reported in the literature. The high complication rate in the postoperative period in group 1 suggests that the underlying pathology is also a factor in legal blindness in the other eye (**Table 3**).

In our study, if the cause of eye loss in patients with permanent legal blindness in one eye was a surgical complication, the possibility of complications during surgery in the other eye was found to be high. For this reason, the right approach may be to prefer the operation of the patient’s other eye, whose first surgery was complicated by an experienced surgeon.

Topical anesthesia is currently the preferred method for cataract surgery [19]. In our study, topical anesthesia was administered to all patients. Due to the low compliance of the patient who was operated on with topical anesthesia, there may be a risk of complicating the first operation [19]. If patients with permanent legal blindness in one eye have low compliance, additional anesthesia (subtenon, peribulbar, retrobulbar, sedation, general anesthesia, etc.) may be another option. It should be noted that these processes may cause additional problems. Ajay et al. [20] reported that the surgeon’s comfort deteriorated, and moderate-to-severe discomfort occurred in 17.2% of cataract cases where anterior subconjunctival anesthesia was applied and 3.6% of cases in which subtenon anesthesia was used. In our study, no such evaluation was performed.

Few studies exist on patients with permanent legal blindness in a single eye. Our study’s limitations are that it was a single-center study with a limited number of patients and that the statistical evaluation was applied to a limited number of patients.

## Conclusion

To our knowledge, this is the first report to compare the rate of complications during and after surgery in patients with pre-existing permanent legal blindness in one eye who underwent cataract surgery and vitreoretinal surgery for retinal detachment in the other eye. When eye loss occurs due to diabetic retinopathy, age-related macular degeneration, corneal leukoma, amblyopia, or glaucoma, the expected complication rate in surgery of the other eye is not different from that in normal eyes. The risk ratio is not significant in the surgery of the eyes of permanently legally blind patients in one eye, which is due to the reasons mentioned above, and it seems to be relatively easy to make a surgical decision in these cases. Nevertheless, it is essential to consider that individuals with permanent legal blindness in one eye due to prior surgery may have an increased risk of complications during surgery in the other eye.
